# Fibromyalgia and Associated Disorders: From Pain to Chronic Suffering, From Subjective Hypersensitivity to Hypersensitivity Syndrome

**DOI:** 10.3389/fmed.2021.666914

**Published:** 2021-07-14

**Authors:** Yves Maugars, Jean-Marie Berthelot, Benoit Le Goff, Christelle Darrieutort-Laffite

**Affiliations:** Rheumatology Department, Nantes University Hospital, Nantes, France

**Keywords:** fibromyalgia, pain, fatigue, diagnosis, psyche, neuro-imaging, hypersensitisation

## Abstract

The concept of fibromyalgia has progressed to achieve a certain consensus regarding the definition of the condition. We summarize what is known in 2020, be it in terms of diagnosis, with the criteria that have changed over the years, or at the level of the psychological profile, via the notions of “catastrophizing” and “coping” and post-traumatic syndrome. The importance of fatigue and sleep disorders is underlined, with the chronological sequence of post-traumatic syndrome, chronic fatigue, and then amplification of the pain and the onset of multiple associated symptoms. The etiopathogenic debate has been enriched thanks to neuro-imaging data to discover the start of the central neurological signature. The many associated symptoms are reanalyzed in the context of so-called sister conditions which form sometimes more or less separate entities, such as chronic fatigue syndrome or restless legs syndrome for example. What these conditions have in common is hypersensitivity, not just to pain, but also to all exteroceptive stimuli, from deep sensitivity in the neuro-vegetative system, the sense organs and certain functions of the central nervous system, to the psychological aspects and sleep control. In summary, it is possible to define fibromyalgia as a cognitive disorder of cortical integration of chronic pain, with amplification of painful and sensory nociception, decrease in the threshold for the perception of pain, and persistence of a stimulus that maintains the process in chronicity. Fibromyalgia is part of a group of chronic hypersensitivity syndromes of central origin, with a very wide range of means of expression.

## Introduction

Fibromyalgia is a very common condition. Its concept was discussed for many years before being as well-defined as it is now. The first false idea was to believe that it was a new condition. Back in the nineteenth century, there were already descriptions that were perfectly typical of fibromyalgia, for example by Beard (1). Then, for most of the twentieth century, the condition was called fibrositis (2). The many subsequent studies led to the statement that there was indeed pain, but on the contrary no sign of inflammation, meaning that the suffix -itis was not appropriate. This was why Hench created the term “fibromyalgia,” this time using the suffix -algia. The term has been used for around 40 years now, in line with the development around the world of anti-pain centers (3). Yunus took up the term in the 1980s, and it remains in use today even though, as we shall see, pain is only one of the components in a complex set of symptoms (4). We will thus use the term fibromyalgia throughout this review. As we shall see, another concept has emerged, led by Yunus, that of neurosensory amplification: hypersensitization.

The typical fibromyalgia profile can be described in just a few words. The patient is a young woman who has suffered from pain, in an intense manner, all over, for several months or years and without any diagnosis being made. More than 9 times out of 10, patients are women, although there is no clear explanation for this. They are more often than not young, in the 20–60-year range. After the age of 60, fibromyalgia is less frequently diagnosed. The prevalence in Minnesota showed a trend of a decrease with the age, respectively, 8.45%/6.02%/3.79% in 21–39/40–59 and over 60+ years groups (5). But only there were only 27.6% responders to a survey which was sent by mail, which is less used by older people. Il is possible that over 60 years old generalized pain, fatigue and sleep disorders could be more easily attributed to osteoarthritis and old age. Before the age of 20, fibromyalgia is also rare, but we are nevertheless seeing fibromyalgia patients who are increasingly young, with a prevalence that remains constant (6). The costs of fibromyalgia are widely under-estimated. A study carried out in the United States compared over 1 year all the costs associated with fibromyalgia with rheumatoid arthritis (7). Globally speaking, there was little difference: $12,000 for fibromyalgia and $14,700 for rheumatoid arthritis. In more detail, although rheumatoid arthritis is associated with high treatment costs, because of biotherapies, we can see that the difference is not as much as all that. On the other hand, fibromyalgia is more costly in other areas, such as imaging, medical biology, and physiotherapy. Although rheumatoid arthritis is a condition with potential joint destruction and deformation, which is not the case in fibromyalgia, there are significantly more days off work for fibromyalgia, with an equivalent degree of invalidity. Fibromyalgia thus has a high annual cost because of its medical nomadism, the requests for multiple and often unnecessary examinations, and finally in the search for a wide range of treatments which are added on despite their poor efficacy. Furthermore, fibromyalgia is 5–10 times more common than rheumatoid arthritis.

We will approach the condition first from a classic point of view, passing from the concept of pain to that of suffering, involving complex psychological integration phenomena. We will then discuss the psychological characteristics, the etiopathogenic aspects and the major input of neuro-imaging. Finally, we will discuss the modern concept of hypersensitization syndrome of central origin.

## Typical Symptoms

The symptomology, like the reason for consulting a doctor, is dominated by pain. It has an essentially lumbar and gluteal, cervical and scapular, dorsal paravertebral axial topography. It predominates above all at the level of the cervico-scapular and lumbar-pelvic belts. A study has compared to a painless group the intensity and location of pain in 5 painful conditions: painful temporomandibular disorder, headache, low back pain, irritable bowel syndrome, and fibromyalgia (8). Fibromyalgia is clearly individualized by whole posterior axial pain and headache (temple, behind eyes, forehead, and inside), and with less pain at feet, arms and thighs. So peripheral pain in the limbs is rarer, although it has been observed in the knees, elbows and hands. The intensity of the pain is significant, often between 6 and 10, with a non-negligible proportion of patients who would put their pain at more than 10 on the pain scale, even though they maintain their regular daily activities (9). Mean evaluation of the pain in fibromyalgia is much more important than for rheumatoid arthritis or lupus, respectively, at 6,0–4,1 and 3,9 in a comparative study (10). The pain can be present for many months, often several years or even decades.

Fibromyalgia pain varies significantly over time, depending on the period, the season, and the activities (11). Certain factors thus aggravate the pain, the most important of which is inactivity (12). A vicious circle then develops: the pain forces patients to stop certain activities, and the consequence of this is even more pain. On the contrary, sport, which is, along with physical activity, an element of choice in the treatment, together with heat and relaxation, are factors that are favorable and on which it is necessary to count. Humidity and cold also have an impact on the pain, as is the case for rheumatic pain (13). Stress is a very clear aggravating factor (14). Alcohol could be a factor that improves the pain, although obviously it is not therapeutics (15). Finally, fatigue is clearly associated with aggravation of the pain, as we shall see.

Fibromyalgia is initially difficult to diagnose because the associated signs can simulate many other conditions (16). Here we can mention for example paresthesia of the upper or lower limbs, which can suggest carpal tunnel syndrome or any other entrapment syndrome or polyneuritis; an acrosyndrome can suggest Raynaud's syndrome; mouth dryness can simulate Gougerot-Sjögren syndrome; feelings of swelling can simulate rheumatoid arthritis or spondyloarthritis or psoriatic arthritis; morning stiffness can simulate polymyalgia rheumatica; headaches can resemble migraines; balance disorders can resemble vertigo; and finally, atypical digestive, urinary, visual and auditory disorders have been reported.

It is also very important to screen for the signs associated with the pain, as they can have both a diagnostic and prognostic value. Sleep disorders are almost constant, of long date, with insomnia or sometimes sleep that feels real but which is not in fact restorative (17). This is how the fatigue takes hold, from the moment the patient wakes up in the morning. The intensity, which is often considerable, confines patients to inactivity and is described as a feeling of total exhaustion. It should be noted that sleep apnea syndrome is associated with fibromyalgia in almost one in three cases, and this should be remembered systematically in obese female patients who snore (18). There can also be muscle fatigue. Other important associated signs should also be screened for, even if they are not reported spontaneously by the patient. These include headaches, in approximately one case in two, anxiety, stress and depression in approximately one case in three, and irritable bowel syndrome, also in one case in three (post).

In contrast with the clinical picture of very intense pain with a wide variety of so-called functional or subjective associated signs noted by the patients, the objective examination is perfectly normal (8). There is no synovitis, no tenosynovitis, which can be verified by ultrasound or MRI, no tendinitis, even though the pain points are more often than not located in the same enthesopathic areas. There is also no objective alteration to muscle strength, even though myalgia can be found on deep palpation, and the neurological examination is normal. There are no significant or specific cutaneous signs either. What can be found on clinical examination are multiple pain points: diffuse, bilateral, and symmetrical, the core of the old ACR diagnostic criteria for fibromyalgia, dating from 1990 (19). They correspond to spontaneous reported pain, above all axial in two belts: the cervical-scapular belt and the lumbopelvic belt, but also at the anterior thoracic level. There are fewer peripheral pain points and the most characteristic are the lateral epicondyles, crow's feet, trochanters, the greater tuberosity of the humerus and the internal supramalleolar muscles. It is possible to use sensors to visualize the allodynia where the pressure is low but where a simple handshake or discreet pinching in the trapezoids is enough to produce significant pain. As we shall see later, any pressure is in fact allodynic and the non-specific points are simply those which are the most sensitive.

## Additional Examinations, Differential Diagnosis

Additional examinations are essential for making a definitive diagnosis (20). Early diagnosis can improve patients satisfaction. Therefore, an initial comprehensive evaluation to exclude other conditions may help patients to realize their conditions and reduce uncertainty. A biological work-up is also essential. The results can vary from one patient to another, and from one doctor to another (21). The results may be normal, thus making it possible to eliminate other differential diagnoses. It is also possible to propose, systematically and as routine, a blood count, sedimentation rate, and C-reactive protein test so as to eliminate an inflammatory condition, protein electrophoresis to eliminate pathologies of the M-spike type or gamma globulin disorders, serum calcium, and phosphorus levels to eliminate hyperparathyroidism or osteomalacia, creatine kinase to make sure not to miss a muscle disease, and thyroid-stimulating hormone is essential for eliminating dysthyroidism. Depending on the context, if chronic inflammatory rheumatism is suspected, the decision can be made to screen for antinuclear antibodies, a rheumatoid factor and anti-CCP antibodies for rheumatoid arthritis, anti-thyroid peroxidase antibodies for Hashimoto's disease, anti-neutrophil cytoplasmic antibodies to make sure vasculitis is not missed, and HLA B27 if spondyloarthritis is suspected. If the progression is semi-recent and can be counted in months, other serological, virological or other tests may be useful, depending on the context. We can also cite the cytomegalovirus, Epstein Barr and herpes viruses, toxoplasmosis, Lyme disease, chikungunya and dengue fever in case of travel to tropical regions, as well as trichinosis and brucellosis which are nevertheless exceptional if there is no travel to countries where these conditions are endemic. Radiology and imaging examinations are said to be normal, though it would be better to say that there are no specific elements. Depending on the degree of painful discomfort, it may be useful to suggest a pelvic X-ray to look at the sacroiliac joints, X-rays of the lumbar, dorsal, and cervical spine to identify any arthrosis or degenerative disc disease, but with non-specific ordinariness, X-rays of the shoulders and hips in search of apatite calcifications, which can also affect both belts, as is predominantly the case with fibromyalgia pain. Finally, an X-ray of the thorax can be justified to diagnose sarcoidosis. Other additional examinations are often requested out of excess, even if it is possible to need imaging to screen for bone or enthesitic lesions; an electromyogram to screen for radiculopathies, entrapment syndrome or polyneuritis; an ultrasound to identify synovitis, tenosynovitis, bursitis or tendinitis; a CT scan or MRI, often axial although degenerative conditions identified in this way are often misleading because they are very common and asymptomatic, such as osteoarthritis, degenerative disc disease and calcifications.

This long list of differential diagnoses, classified by organ (Table 1) summarizes the work of the diagnostician who aims to screen for and eliminate all them all, first of all clinically of course, and with the help of any appropriate additional examinations. It is possible to highlight the four pathologies that pose the greatest diagnostic difficulties in everyday practice: osteoarthritis, spondyloarthritis, dysthyroidism, and apatite-induced arthropathy. Muscle, bone, neurological and endocrinal workups can complete this diagnostic inquiry. But what really matters is that the practitioner has acquired a strong degree of confidence with the diagnosis of fibromyalgia, as this will also allow the patient to obtain the same diagnostic confidence. It has been shown even though the practitioner may request fewer additional examinations, the patient was less stressed and felt better (22).

**Table 1:** List of differential diagnoses to be eliminated by the rheumatologist in cases of diffuse pain.

Muscle pathologies	Myositis Myopathy
Joint pathologies	Osteoarthritis Spondyloarthritis Rheumatoid arthritis Other form of vasculitis
Bone pathologies	Osteomalacia Fracturary osteoporosis Secondary bone localizations
Neurological pathologies	Myelitis Polyradiculoneuritis/polyneuritis Amyotrophic lateral sclerosis
Endocrinal pathologies	Hypothyroidism Hyperthyroidism Hyperparathyroidism
Viral pathologies	Cytomegalovirus Epstein Barr Herpes virus Chikungunya Dengue
Miscellaneous	Apatite-induced rheumatism Sarcoidosis Chronic Lyme disease Chronic toxoplasmosis Trichinosis Chronic brucellosis

## Psychologic Profile

Understanding the psychological profile of fibromyalgia patients is one of the keys to better understanding the condition. The first question that needs to be asked is very simple: is fibromyalgia a matter of rheumatology, psychiatry, or algology? As we have already seen, rheumatologists play a key role at the diagnostic level. The psychiatry profile of fibromyalgia has been clearly defined, and it conditions how it will be dealt with subsequently. Finally, algology is a discipline involving natural management of this pain, with a multi-disciplinary approach given this level of multi-dimensional complexity.

From the concept of neurasthenia to that of fibromyalgia, a great many things have been said about the psyche of fibromyalgia patients. First of all, it is once again necessary to lay to rest the stigma of fibromyalgia as some kind of hysteria or pithiatic condition. There are, of course, fibromyalgia conversion disorders, but no more than in a control population (23). Let us now turn to more classic psychological profiles: depression, anxiety, stress, cognitive disorders, plus a few words about sexuality, before talking at greater length about the notions of coping, resilience, catastrophizing, and post-traumatic syndrome. It should be noted that all these psychological elements are highly variable from one fibromyalgia patient to another, with a very clear correlation between the intensity of the pain and the psychological profile.

Depression is a classic disorder found in ~1 patient in 4, which is 2–3 times higher than in a control population (24). The main question is to know whether this depression is primary or secondary. Its importance has almost certainly been overestimated, and we can thus consider it normal that a woman who has been in pain for several years, with multiple questions and no reliable treatment response, can have a certain degree of relational depression. It should be noted that the depression is more marked when the pain is intense, and that depressed fibromyalgia patients are generally younger, with more pain, more sleep disorders, less social satisfaction, less well-being and a greater need for assistance (24).

The same observation can be made for anxiety and stress: there is the same prevalence of approximately one third, the same probably reactional origin, the same overestimated importance because it is expressed with insistence by fibromyalgia patients, and the same correlation between pain, anxiety, and stress (25).

Cognition is undoubtedly altered in fibromyalgia patients, whether it is attention, reasoning, memory, learning, perception, or recognition (26). The cognition disorders are associated with the symptom of fatigue, which is significant, with even sometimes exhaustion. This poor cognition is not only self-reported, but also objective when evaluated with neuropsychological measurement. Fibromyalgia patients have up to half of the short-term or long-term memory when compared to an age-matched control group (27). The cognitive impairments are correlated negatively with the degree of pain, but also with affective factors, as catastrophizing, low self-esteem and alexithymia (28).

Surveys have clearly shown that there are sexual disorders in fibromyalgia patients (29). With this, there are two approaches to adopt. The first is that although there is less sexual satisfaction, it is hard to imagine how anything else could be the case in these patients who not only endure diffuse pain, but also endure pain that is exacerbated by the slightest touch, without forgetting the gynecological points which are not spared by the allodynia. However, fibromyalgia patients have little or no alteration at the level of their desire or pleasure, even though there is no consensus in the studies on this subject. A second approach is also important. Sexual abuse can be found in the past history of 20–50% of fibromyalgia patients, either in childhood or as adults. The relative risk of finding sexual abuse in relation to a control group is 2.5–3.1 for fibromyalgia patients (30). This notion needs to be placed in the context of post-traumatic syndromes.

The term “coping” is often used, from “to cope,” which comes from the French *couper*, to cut, separate, hit. And we find that in expressions like “cut to the quick.” The meaning of coping, though, can be summarized in this definition: all the permanently changing cognitive and behavioral efforts that an individual uses to respond to specific internal and/or external demands, evaluated as very high and going beyond his or her adaptive resources (31). The term is associated with the notion of resilience developed by Boris Cyrulnik. In certain people, the default “coping” strategy, with difficulty adapting to a complex situation, will lead to a psychological disorder: dissociation, sensitization, safety attitude, avoidance, or self-medication. We can immediately see the association with a causal factor, the consequence of a post-traumatic stress syndrome. The psychometric evaluation can be made using the Chronic Pain Coping Inventory-42 (CPCI-42) or the Coping Strategy Questionnaire (CSQ). In fibromyalgia patients, the coping is deficient. Thus, in difficult situations, and here we can cite for example a bombing, sexual abuse, a natural catastrophe, war, or holocaust. But much less extreme events can be traumatic in these deficient coping patients who develop fibromyalgia. They will not be able to find solutions that allow them to move forwards, and turn in circles around the traumatic event.

The second important term to define the profile of fibromyalgia is “catastrophizing.” This is first and foremost a pessimistic attitude, negativism, alarmism, someone who sees everything as black and always sees the worst in a situation and is tied to disaster (31). It can be evaluated using the Pain Catastrophizing Scale, or the Sullivan Scale. The notion of trauma, in the broadest sense of the term, is once again significant. The same catastrophe situations that generate “coping” can be associated with the notion of “catastrophizing,” as for example in climate changes, conflicts and wars, natural catastrophes and possibly viral pandemic in the future.

Catastrophizing is an important pattern in fibromyalgia, clearly associated with pain intensity, provoked pain (9). Nevertheless, functional magnetic resonance imaging did not found any association between the assessment of an experimental provoked pain and catastrophizing, as well as for anxiety or depressive symptoms (32). There were no correlations with the duration of the fibromyalgia. If the pain sensitivity is increased in fibromyalgia, its neural mechanism seems in part different from the negative mood affects, as depression, anxiety and catastrophizing. But high catastrophizing in fibromyalgia could take part in a decrease of the pain inhibition which occur during distraction cognitive tasks, in a model of thermal stimuli with functional MRi assessment (33). This mechanism could contribute to explain the pain and the other symptoms persistence, with the need of interventions to reduce catastrophizing, as cognitive behavioral therapy.

Psychological trauma plays a very well-defined role in the genesis of fibromyalgia, with prospective follow-up studies such as the one by Lawrence-Wolff. In this study, on American Gulf War veterans, 40% had been in a war situation, and the diagnosis of fibromyalgia went from 2 to 8% on their return, with a quarter presenting post-traumatic syndrome (34). Patients at risk had already suffered from pain in the past (relative risk × 2.4), but above all catastrophizing (relative risk × 11). Traumatic events can also be physical, associated with a secondary psychological impact. A prospective, 2-year follow-up study on patients admitted to the Emergency Room after a car accident in London and Toronto is the perfect illustration (35). It was possible to identify three groups: 383 serious accidents but with no cervical impact; 224 accidents involving cervical trauma; and 643 minor accidents. None of these patients had suffered pain before. In relation to the reference group—minor accidents—the traffic accidents developed almost 5 times more cases of fibromyalgia at 2 years, with a relative risk of 5.2, and a rate of 3% when there was a cervical impact, with a relative risk of 8.4. We can see here the important role played by trauma and the cervical location, which is a source of anxiety given the potential seriousness.

Professional problems are important, with repeated periods of sick leave which often inspire a lack of understanding given the organic mildness. But the long duration of the symptomatology, plus the personal, professional, and family desocialization can deserve a better attention (36). Associations for fibromyalgia patients have thus played a part in improving understanding of the condition in both the general public and the medical profession, and thus recognition of an affection that is not “made up.” Alongside these positive actions, requests for classification of the condition as chronic long-term illness and handicapped status for all, the quest for a miracle drug or a perfect diet that does not exist, as well as belief in sometimes doubtful theories developed by some, all still have a negative impact on the credibility of fibromyalgia patients, who are nevertheless barely affected by this contestation. Finally, a precise psychological diagnosis and the psycho-social impact are important to be evaluated, in order to offer adapted psychological interventions, which are a major point of the therapeutic approach (37).

## Diagnostic and Evaluation Criteria, Prognosis Factors

The FIRST questionnaire makes it possible to detect a target fibromyalgia patient population (38). These 6 statements are expressed as being the most specific for the experience of fibromyalgia patients (Table 2). If patients recognize themselves in at least five of them, there is a 90% chance that they have fibromyalgia.

**Table 2:** FIRST questionnaire to identify fibromyalgia within a population.

**6 selected questions**
1. My pain is localized all over my body
2. My pain is accompanied by permanent general fatigue
3. My pain is like burning, electrical discharges, or cramps
4. My pain is accompanied by abnormal sensations, such as tingling, pins and needles or numbness, all over my body
5. My pain is accompanied by other health problems such as digestive disorders, urinary tract problems, headaches, or restless legs
6. My pain has a significant impact on my life: in particular, on my sleep, my ability to concentrate, giving me a feeling of being in slow motion

*FIRST positive if **≥ 5/6** of the items present*.

*Sensitivity 90% and Specificity 86%*.

After the initial diagnostic criteria dating back to 1990 (19), with a low degree of specificity and putting the highlight on pain points, Yunus and Wolff developed new diagnostic criteria in 2010, revised in 2016 (39, 40). The 2010 diagnostic criteria were composed of 2 chapters. One listed the spontaneously painful points, the other the associated symptoms. The 19 pain points were reduced to 15 in 2016, but nevertheless they still focused much too heavily on peripheral pain (12 of the points) in relation to axial pain (3 points), when the hierarchy in relation to their respective importance is the opposite (Table 3). The associated symptoms are divided into four chapters: fatigue, non-restorative sleep, cognitive disorders, and other associated symptoms. They remain rather unspecific. The diagnosis is confirmed on the one hand if there are at least seven pain points with an associated symptom score of at least five. Thus, for example, pain in the shoulders, hip, and spine, combined with fatigue and fragile sleep patterns associated with some attention deficit disorders would satisfy the criteria. It is nevertheless easy to see how a differential diagnosis might be missed with diffuse pain of other origins. The notion of absence of another diagnosis is thus very important. The 2016 criteria, however, added that there are sometimes associated, making the diagnosis extremely difficult. The 2nd diagnostic approach has very few pain points, essentially the spine, and a wide range of associated symptoms. The 2016 revision requires at least a 4th pain point (39). These diagnostic criteria can be used not only in studies, but also when one asks questions about a sure diagnosis of fibromyalgia because they provide sensitivity and diagnostic specificity values of 80–90%, which is very acceptable.

**Table 3:** ACR 2010 diagnostic criteria, revised in 2016.

Widespread pain index (WPI) (pain > 3 months) (circle the areas that were painful during the past 7 days)				
	Neck			
Right shoulder				Left shoulder
Right upper arm				Left upper arm
Right forearm				Left forearm
	Upper back			
Right hip/buttock				Left hip/buttock
Right upper leg				Left upper leg
Right lower leg				Left lower leg
	Lower back			
Calculation of the number of painful areas: TOTAL 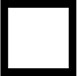 …out of 15				
Severity of the symptoms (SS) during the past 6 months [from 0 (none) to 3 (severe)]				
Fatigue	0	1	2	3
Non-restorative sleep	0	1	2	3
Cognitive disorders	0	1	2	3
Severity of the associated symptoms	0	1	2	3
Calculation of the severity of the symptoms: TOTAL 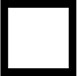 …out of 12				
Positive ACR diagnosis				
	if	WPI ≥ 7 and SS ≥ 5		
or	if	WPI 4–6 and SS ≥ 9		
and	if absence of other diagnosis			

*But sometimes a possibility of an associated condition*.

*Sensitivity 88% and Specificity 81%*.

*To be completed by the doctor*.

In studies that assess fibromyalgia, in addition to pain and fatigue type visual analog scales, the FIQ questionnaire, revised in 2009, makes it possible to obtain an overall approach on the progression of the fibromyalgia (41, 42). It has thus become essential in the various studies assessing fibromyalgia. Ten per cent of the questionnaire is associated with function, 20% with the number of days without activity, and 70% for difficulties encountered in professional and everyday life. Fibromyalgia is located at scores of around 50–70 out of 100, depending on the severity.

The prognosis factors are associated with the severity of the symptomatology, be it the fatigue, pain, associated signs, or the global index, the FIQ (31). But the psychological factors are also prognostic, be it the depression, anxiety, stress, or realm of catastrophizing (25). There is no over-risk of mortality in fibromyalgia (43). As for the progression in the mid-term, there is little to no change at 1 or 2 years (44). The long- and very long-term progression is poorly known, but it can be noted that there are few or even no cases of fibromyalgia after the age of 70 years, even if the condition has existed for a long time. A small cohort monitored at 26 years revealed that 11% of the fibromyalgia cases had been cured and 23% had remission lasting more than 1 year (45). But a recently published study revealed that the risk of suicide is far from negligible. A major meta-analysis of almost 400,000 fibromyalgia patients very clearly revealed more suicidal thoughts, with a relative risk multiplied by 9.1, and suicide attempts, with a relative risk multiplied by 3.1 (36). Of the other risk factors, we can mention professional status, the severity of the fibromyalgia (whether via the VAS for pain or the FIQ), chronicity, obesity, and addiction to opioids. We cannot stress the last factor enough as it is one of the “big affairs” in drug prescriptions in recent years, with, it should be remembered, a mortality rate of 30,000 people a year in the United States. Finally, there is always the psychology of these patients, with depression, anxiety, mental health, and sleep disorders. Fibromyalgia should thus be taken seriously by screening in severe cases for these risk factors of suicide, and screening for profound depression associated with the severe cases, plus addiction to opioids or products such as ketamine which can disinhibit and provoke a dissociative effect.

## Etiopathogenic Debate: Pain, Sleep Disorders, and Fatigue

Here are a few questions for which we can try and provide some elements of response. What is the reality of the pain, with peripheral explorations and the role of neurotransmitters yet to be defined? Where does the pain come from, with notions of hyperalgesia and allodynia, the notion of pain thresholds and pain amplification? What is the role of fatigue and sleep disorders, and what possible role of induction do they have?

Substance P and glutamate, secreted by nociceptive neuronal fibers, have been the subject of unambiguous studies, with high concentrations in the CSF of fibromyalgia patients in relation to controls, in a non-specific manner, and with normal serum concentrations (46). The same is true for neurotrophins of the NGF or BDNF type (47). Enkephalins may be good candidates. Naloxone has given disappointing results, and may provoke diffuse pain, like morphine (48). Endorphin concentrations in the CSF of fibromyalgia patients remain normal, and as we have already seen, morphine is a disappointing treatment for fibromyalgia pain (49). Similarly, a study using the PET scan with Carfentanyl, which binds to endogenous μ-opioid receptors in the regions of the central nervous system devoted to pain, found a decrease in the signal in 17 fibromyalgia patients in relation to 17 controls (50). There is thus an inverse correlation between the intensity of the pain and binding at the level of the nucleus accubens, stratum and cingulate cortex. The hypersensitivity to pain in fibromyalgia patients is in contrast to diminished activity in the central nervous system and explains the disappointing results of opioids when treating fibromyalgia pain. In addition, the risk is an increase in doses which can lead to addiction and toxicity, with all the dramatic consequences and mortality that that implies.

Serotonin could have been the key neurotransmitter in fibromyalgia. It effectively modulates nociceptive information at the supramedullary level, it regulates sleep and is associated with the presence of slow waves. There are different sites and brain concentrations in men and women, and it is modulated by estrogens (51). Thus, in fibromyalgia, concentrations of serum tryptophan, the precursor for serotonin, are decreased. There is polymorphism in the serotonin receptors. Serum serotonin concentrations are inversely correlated with the severity of fibromyalgia, and concentrations of 5 HIAA in the CSF, the metabolite of serotonin, are decreased. However, the serotonin re-uptake inhibitors are not every efficient on the pain of fibromyalgia. That is more the role of serotonin and noradrenaline re-uptake inhibitors. The neurotransmitter approach and fibromyalgia can only be envisaged by associating several together, not just serotonin, but also GABA, dopamine, and noradrenaline (51).

The allodynia is not limited to the standard pain points of fibromyalgia. The pressure of an armband on the muscles in the arm is thus more painful in fibromyalgia patients, with a pain threshold that is ~20–30% lower than in a control group (52). In addition, allodynia is the rule in fibromyalgia 7 times out of 10, whereas it is rare in arthrosis, rheumatoid arthritis and in controls with no **illness**. This hypersensitivity is correlated with the parameters of progression of fibromyalgia. Hyperalgesia and allodynia are thus generalized, associated with an alteration to the pain threshold. But this decrease in the threshold for pain is not only related to painful pressure. A study also tested, in addition to pressure pain, heat, cold and stings (53). The decrease in the receptiveness threshold was around 20%, except for cold, where it was actually more than 100%, during or after the stimulus.

This mechanism can be explained by the fact that in normal physiological situations, whether we are standing, sitting, walking or even lying down, we always have a certain muscle tone, a certain tension in our tendons and muscles, and stimulation of the receptors, even at room temperature. There is thus a certain amount of nociception which is activated and stimulated permanently. But when this nociceptive signal level is low, the brain integrates the signals as normal and does not emit any information of the pain type. Among these hypotheses, we can point the finger either centrally, with the collapse of the pain threshold, or an amplification mechanism for this normal signal, or alteration to the retrocontrol mechanisms of the inhibitor system. In any case, the result is the same, with amplification of the pain signal felt, which can be illustrated as the volume control button on a music system. Finally, the response time for pain is lengthened, just like the duration of the response felt to pain. The same amplification mechanism can be felt after sensory stimuli with quantitative measurements in functional MRI, whether the stimuli are visual, auditory, temperature or sensory (54). The intensity of the signal is correlated with the intensity of the pain and the functional handicap induced.

Along with the pain, fatigue is the other major element in the clinical picture that must be highlighted in fibromyalgia. One study illustrates this particularly well: 87 adults who practiced sport and had no sleep disorder were randomized into four groups: controls, restricted exercise, restricted sleep or restricted exercise and sleep (55). By the 10th day, symptoms equivalent to those found in fibromyalgia started to appear in restricted sleep groups: pain, major fatigue, and thymus and cognition disorders. It should be noted that exercise had a certain inhibitory effect in men, and that fatigue played a preponderant role in women. In personal practice, everyone has been able to observe that after periods of troubled sleep, or a sleepless night, or a viral syndrome, there is generalized soreness or aching, associated with the induced fatigue.

Sleep disorders are thus almost always mentioned. They are real, as observed by polysomnography (17). It is possible to observe decreased sleep duration, altered sleep quality with more phase 1 and fewer slow waves, and an increase in the number of nocturnal wakings. These anomalies can be quantified using the Pittsburg Sleep Quality index (56). There is real dissociation between the sentiment of sleep disorders and the objective data. What remains is that while this non-restorative sleep certainly plays a role in understanding the physiopathogenesis of fibromyalgia, it has not so far resulted in drug therapy measures that are genuinely effective, with mitigated results with melatonin and disappointing results with agomelatine, a melatonin agonist (57, 58).

**Figure 1:**
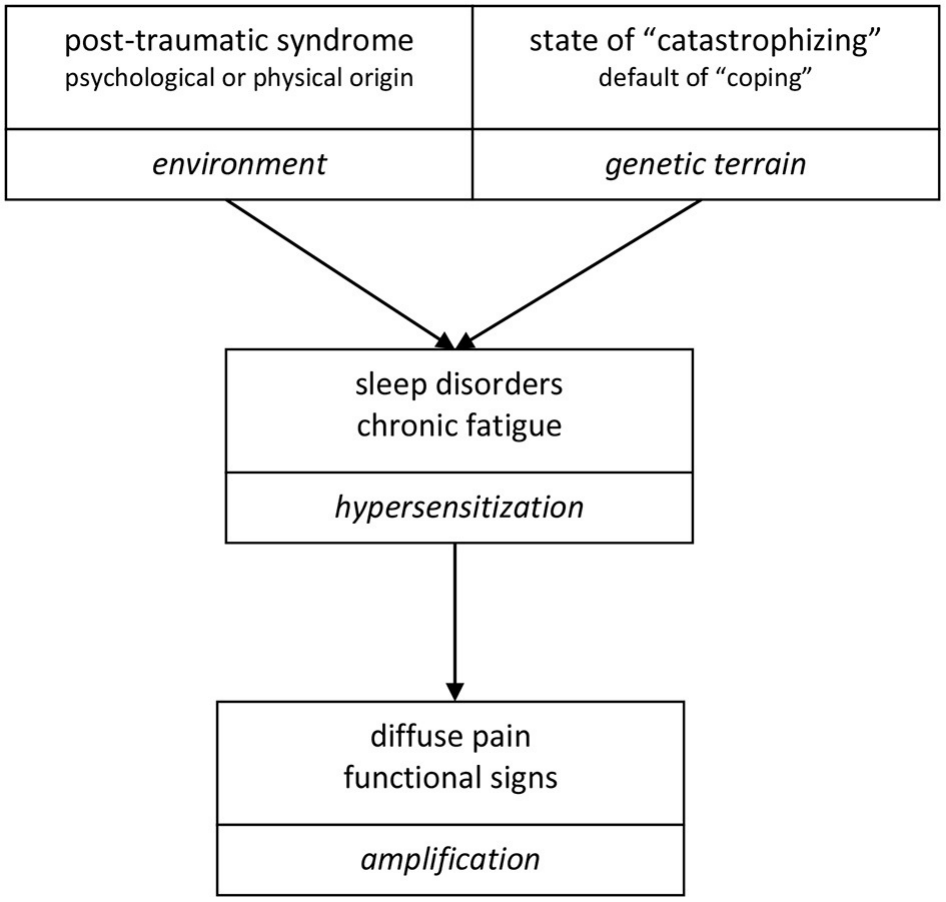
Respective role played over time of the psyche, sleep disorders, fatigue, pain, and sensory functional disorders: kinetic analysis of the events occurring in the life of a fibromyalgia patient. The traumatic psychological events are old, or even dating back to childhood. The sleep disorders are secondary and quickly associated with a state of fatigue, which precede often by several years the chronic pain state and associated functional signs, in the sequence illustrated below.

The kinetic analysis of events is important to better understand their role in the etiopathogenesis of fibromyalgia (Figure 1). In the follow-up to post-traumatic syndrome, regardless of the trauma, in certain fields particularly associated with the persistence and non-resolution of problems, with a more or less catastrophist or negative vision of things, the psychological disorders induced are associated with sleep disorders. The result is chronic fatigue, associated with hyperreactivity and overall hypersensitization, with amplification phenomena in the sensory signals that are expressed loudly by persistent, invalidating pain at rest. All feelings are hypertrophied, regardless of the sensory or psychological nature. We can observe that chronic fatigue precedes, often by several years, the onset of the pain and all the associated functional signs.

## Etiopathogenic Debate: Neuro-Imaging, Small Fiber Neuropathy, and Epigenetics

Modern imaging methods, as PET scan combined with functional scintigraphy or functional MRI, have been possible to identify the structures involved in central integration of pain. The well-known entry point for pain is the thalamus. It sends multiple connections toward the cingulate cortex, the sensory cortex, and the insula. All these regions send information on sensory discrimination, intensity, and the affective and psychological aspect of the pain. The diagram of these interconnections is of still poorly understood complexity, in a global dynamic process, with input, output, integration, memorization and modulation that we are only just starting to understand (59). Functional brain localizations have been identified for the catastrophizing, coping and depression processes, although there is no correlation between these identified psychological factors and the measures made in functional MRI and hyperalgesia (60). Prefrontal integration of pain is associated with catastrophizing. Pain is thus modulated in relation to fears, beliefs, and isolation, with integration defects.

The volume of cortical gray matter has been described as decreased in fibromyalgia (61). However, it cannot be explained by a neurodegeneration, with an upregulation of the GABA_A_ receptors in MRI imaging, which could reflect an increase in neuronal matter. Throughout a person's life, neurons have the ability to modify their connectivity and activity in relation to our environment, our epigenetics, and also in relation to our genetics. The same is true with regard to pain (62). The number of neurons involved in pain is <5%, but, in situations of chronic pain, this number rises to 15–25%. Many other cortical regions are involved in modulating the integration of pain. It has been observed that it is necessary to make use of this neuropathic pain and circumvent the neuronal neo-circuits that amplify the pain and cause it to last over time.

In 2017, Lopez-Sola published a definitive article with regard to the search for a real, measurable diagnostic signature in neuro-imaging with functional MRI (63). When a peripheral signal is emitted, it is possible to measure it at the level of certain electively involved regions. But what should we be measuring, given that it has to be something quantitative? A photo of mountains illustrates the results obtained in functional MRI, with peaks and valleys between the different regions measured. It was decided to quantify the signal by capping the activity peaks, with a reproducible manner. The study included 37 right-handed fibromyalgia patients vs. 35 controls paired for age, sex, and socio-educational level. Functional MRI was carried out after nociceptive stimulation to pressure of the thumb, and after neurosensory, visual, auditory and tactile stimulation. Hyperalgesia was found in the fibromyalgia patients in relation to the controls, with amplification of the pain signal of around 50%. A statistical mediation model made it possible to verify that the quantitative measurements in functional MRI truly reflected the variations in pain and unpleasant feelings experienced by the patients. In the first stage, a functional MRI was carried out after nociception with pressure at 4.5 kg/cm^2^. It was thus possible to measure the signal in the regions generally involved in the reception of the nociception of pain, whether this was at the level of the thalamus, somato-sensory cortex, insula, operculum, or anterior cingulate cortex. These measurements were also carried out in the neighboring regions. An increase was observed in the central signal in these regions in relation to the controls, corresponding to an increase of one third of the pressure if the pressure went from 4.5 to 6 kg/cm^2^ in the controls. The ability to make the fibromyalgia—control distinction, as a classification, was 68%. The same methodology was assessed by means of functional MRI at the level of the regions of the brain involved in modulating nociception: the perigenual posterior cingulate cortex, paracentral lobule and precuneus. It was thus possible to study the sympathetic, affective, self-referential, decisional, and sociocultural valences of pain. The difference between the fibromyalgia patients and the controls was very clear, and even more discriminating. The classification power of this second method was 71% between the controls and fibromyalgia patients. A 3rd stage, still following stimulus via painful pressure, measured the weight of the three representative regions by evaluating their ability to distinguish controls from fibromyalgia patients, as much on the positive signals as on the negative ones. Once again, it was possible to make the distinction and a classification at 70% between the two populations. The 4th stage was carried out following sensory stimulus. This stage measured the weight of four representative regions by assessing their ability to distinguish controls from fibromyalgia patients, as much on positive signals as on negative ones, with regard to visual, auditory, and tactile stimuli. The classification capacity was even better, at 89%. Finally, if all four of these stages were combined, the respective regions for the nociception of pain, the weight of the representative regions for pain, and the sensory stimulation, the static analysis revealed a classification capacity of 93%, distinguishing very well the fibromyalgia patients from the control population, with sensitivity of 92% and specificity of 94%. Associations were found between the increase in the brain signal of fibromyalgia patients in functional MRI and pain, sensory stimuli, the FIQ and depression. On the contrary, no association was found between the duration of progression of the fibromyalgia, and the consumption of antidepressants or anxiolytic drugs. There remain many other fields of investigation using this technique with, as a perspective: distinguishing fibromyalgia patients from other forms of chronic pain, assessing the differences between the various hypersensitization syndromes, studying the localizations associated with fatigue, post-traumatic syndrome and coping, and their respective roles, bearing in mind that depression and anxiety do not seem to play a preponderant role, and finally, to find a simple method for assessing fibromyalgia patients with functional MRI with diagnostic and prognostic aims. As we have seen, neuro-imaging has allowed us to make progress in the concept of fibromyalgia as a cognitive disorder of cortical integration of chronic pain. There is thus amplification of the pain and sensory nociception signal, a decrease in the pain perception threshold, and persistence of the stimulus that maintains the process in chronicity.

A small fiber neuropathy has been reported with high prevalence in fibromyalgia. In a recent metaanalysis, a skin biopsy was positive in 45% of 176 patients (64). The diagnosis can be assessed with corneal confocal microscopy with even a better sensitivity. However, these findings are not at all specific, found in many pathologies, such as metabolic diseases, infectious diseases, chronic inflammatory rheumatism, toxic substances and genetic diseases. Finally, there is no correlation between this small fiber neuropathy and the somatosensory system function, so it does not seems to play a role in the pathogenesis of the fibromyalgia (65).

Epigenetics is also a new field of investigation in fibromyalgia (66). DNA methylation and miRNA expression are modified in fibromyalgia, with a role for environmental factors, such as stress, traumatism, sleep disturbance. While the correlations with symptoms are still weak, these explorations are only just beginning, and they could be of interest in the future for a better understanding of the diagnosis, persistence and treatment of fibromyalgia.

## The Polymorphism of Pain

The pain of fibromyalgia is polymorphous and very rich: hyperalgesia, allodynia, hyperpathia, diffuse and variable pain, paresthesia and dysesthesia, with a wide range of varied feelings, including pseudo-algodystrophy, burning, pseudo-arthritis type swelling, tingling, numbness, stinging, pseudo-neurogenic, pseudo-entrapment, and pseudo-muscular or abdominal cramps, spasms and tightness with digestive, vesical, thoracic and gynecological symptomatology. The pain of fibromyalgia can affect every structure in the musculoskeletal system, including the tendons, ligaments, entheses, muscles, joints at the level of any region, above all axial and spinal, but also peripheral, as well as dental, temporomandibular and thoracic for example. Finally, the pain of fibromyalgia can also affect the deep organs, with pain that can be digestive, with epigastralgia and reflux, pseudo-colitis, pelvic pain, vesical pain, headaches, with all the symptomatology contrasting with negative or non-significant additional examinations (67).

There is thus no specificity in the pain of fibromyalgia. Fibromyalgia can be perfectly in adequation with the evaluation scores for other specific pathologies, such as the DN4 for neuropathic disorders, and the BASFI and BASDAI for spondyloarthritis, for example (68). Many diagnostic criteria can be assessed positively in fibromyalgia, such as those for rheumatoid arthritis, but above all for spondyloarthritis, with SSAS criteria that are often positive and, for the latter, a diagnostic problem that remains difficult to solve between female spondyloarthritis and fibromyalgia (69).

Fibromyalgia is sometimes used as a catch-all term to label any condition that is painful but that is not fully understood. And this naturally depends on the rheumatological culture, whether or not we fully master the diagnostic elements, for spondyloarthritis, apatite-induced rheumatism, osteoarthritis, and fibromyalgia. It also depends on the rigor in the initial workup, sometimes skeletal, sometimes even an enormous case file to wade through, with five MRI and three CAT scans, but no thyroid-stimulating hormone. One must therefore be careful not to blindly trust fibromyalgia diagnoses made too easily and in excess. On the contrary, fibromyalgia is still sometimes denied—< 20 years ago, it is true—but still there are colleagues who believe that something that is not well-understood cannot exist, in a sort of exacerbated hyper-rigorism and scientism. There are still also incorrect opinions, classing the condition as hysteria or pithiatic, with a certain amount of ignorance and reductionism of the complexity of the psychiatry. These attitudes still lead to insufficient diagnoses, medical nomadism, and high costs for fibromyalgia.

## The Associated Symptoms, Other Similar Pathologies, Associated Pathologies

Yunus has put together a very long list of the symptoms associated with fibromyalgia pain (70). We can classify them in three groups: the very common, the common and the less common. At the top of the list of very common associations we find chronic fatigue. This symptom can even make us review the diagnosis if it is absent. It is increased when activities are stopped and decreased with exercise. It is correlated with depression, sleep disorders, the intensity of the pain and hyperalgesia. It is also very clearly inter-related with chronic fatigue syndrome, a “sister” **condition**. Sleep disorders are also almost constant, with difficulty falling asleep, non-restorative sleep and light sleeping, and frequent waking. They are associated with emotional disorders, gastro-esophageal reflux, loss of urine and dyspnea, and may be related to sympathetic hyperactivity.

Among the common symptoms that are associated in about one case in two, paresthesia and dysesthesia are correlated with the pain, but not with psychological disorders. They can resemble a more neurological picture such as multiple sclerosis or amyotrophic lateral sclerosis, or a pseudo-entrapment syndrome. Feeling of bloating is common. Headaches resemble migraine, and neurological investigations remain negative. The dizziness is not rotary, with ear explorations also negative.

Of the less common associations, found in roughly one in three cases, we can note dryness in the mucosal membranes, particularly in the mouth, and in the absence of any iatrogenicity, with negative explorations, which sometimes be led to perform a biopsy of the saliva glands; cold extremities, and once again the absence of any iatrogenicity and negative explorations, particularly Doppler; dysmenorrhea, with pelvic pain and sometimes headaches, and once again negative explorations; tinnitus with negative ear explorations; and finally cognitive disorders such as attention deficit, memory or information processing disorders specific to chronic fatigue.

There are also pathologies, often known as functional, that remain close to fibromyalgia (66). We can highlight chronic fatigue syndrome, also similar to “burn out,” which is a very common privileged association, with major, unexplained fatigue as the main common point. Algo-dysfunctional syndrome of the manducatory tract, accompanied by negative stomatological examinations, is also very common. Restless legs syndrome, in one in 3 cases, is associated with abnormal nocturnal movements. Tension headaches, caused by abnormal tension, can be the subject of a request for a brain MRI, which is without particularity. Irritable bowel syndrome, with bloating and alternating diarrhea and constipation, produces a normal coloscopy and presents with many similarities with fibromyalgia, as it also combines headaches and chronic fatigue; it can be treated with duloxetine, like fibromyalgia. Myofascial pain syndrome is accompanied by regional pain with trigger points of a functional nature. Chemical exposure syndrome is a mini-psychosis based on exposure to a supposedly toxic product or a product supposed to be present, combining headaches, nausea, dizziness and pain. It is also possible to make connections with Gulf War syndrome, particularly with the fear of having been exposed to radioactive products even though the in-depth studies carried out have not found any particular cause for the extremely rich and varied functional symptomatology. Pelvic fibromyalgia and the eight pelvic pain points described has led to reports of surgeons performing excessive numbers of unexpected hysterectomies on the basis of the pain alone, even though the other examinations were negative. At the level of the spine and thorax, we can note neck trauma syndrome following a whiplash injury—the diagnosis of a mild sprain is made after negative imaging results yet the pain, which generally lasts for a few weeks, instead lasts for several months or even years. Certain forms of chronic refractory lower back pain are also considered to be the equivalent of fibromyalgia, often following an effective spinal episode but that lasts for months or even years. Tietze and Cyriax syndromes associate sterno-costal pain and slipped ribs, which again last for months and months, without ever being able to find any organic condition.

Finally, all these “sister pathologies” to fibromyalgia can in fact be associated with each other (57). Fibromyalgia can be associated with all of them. Chronic fatigue syndrome can too, with irritable bowel syndrome, tension headaches, Costen's syndrome. Irritable bowel syndrome can be associated with fibromyalgia and chronic fatigue syndrome, but also with tension headaches and pelvic fibromyalgia. Migraines can be associated with restless legs syndrome. And chemical exposure syndrome can be associated with chronic fatigue syndrome and irritable bowel syndrome.

Other organic pathologies have a privileged association with fibromyalgia. The first of which is feminine spondyloarthritis, which as we have seen is a difficult differential diagnosis (69). Female spondyloarthritis has a mean delay of diagnosis between 7 and 9 years, despite the technical means at our disposal, such as MRI. The condition results in little stiffness from a clinical or radiological point of view, and there is a very similar symptomatology—axial—often with a nocturnal pain and chronic fatigue. This association is found in 10–30% of cases of spondyloarthritis (69, 71). It is necessary to determine what proportion of the symptoms can be attributed to each of the two pathologies. Care must be taken regarding the use of biotherapies as they are not relevant if fibromyalgia predominates, even though the more the spondyloarthritis is in flare-up phase, the more the fibromyalgia will be painful. It is also necessary to remember this if biotherapy fails in a case of spondyloarthritis. Fibromyalgia amplifies chronic pain and is a factor in its persistence of chronic painful osteoarthritis of the spine, particularly if there are multiple localizations, cervical and lumbar. It could be a difficult differential diagnosis in women because the symptomatology is similar. In one in three cases of fibromyalgia, Gougerot-Sjögren syndrome is associated with pain that must be differentiated from genuine inflammatory myositis. Autoimmune thyroiditis, or Hashimoto's disease, is rarer, but is also associated in roughly one in three cases with fibromyalgia. Rheumatoid arthritis is less commonly associated with fibromyalgia—roughly one in ten cases. We can also note the role played by stress, depression, and “catastrophizing.” Disseminated lupus erythematosus is associated in roughly 10–20% of fibromyalgia cases, and once again, the fibromyalgia pain must be dissociated from the genuine inflammatory myositis.

## Hypersensitization Syndrome of Central Origin and Etiopathogenic Hypotheses

**Figure 2:**
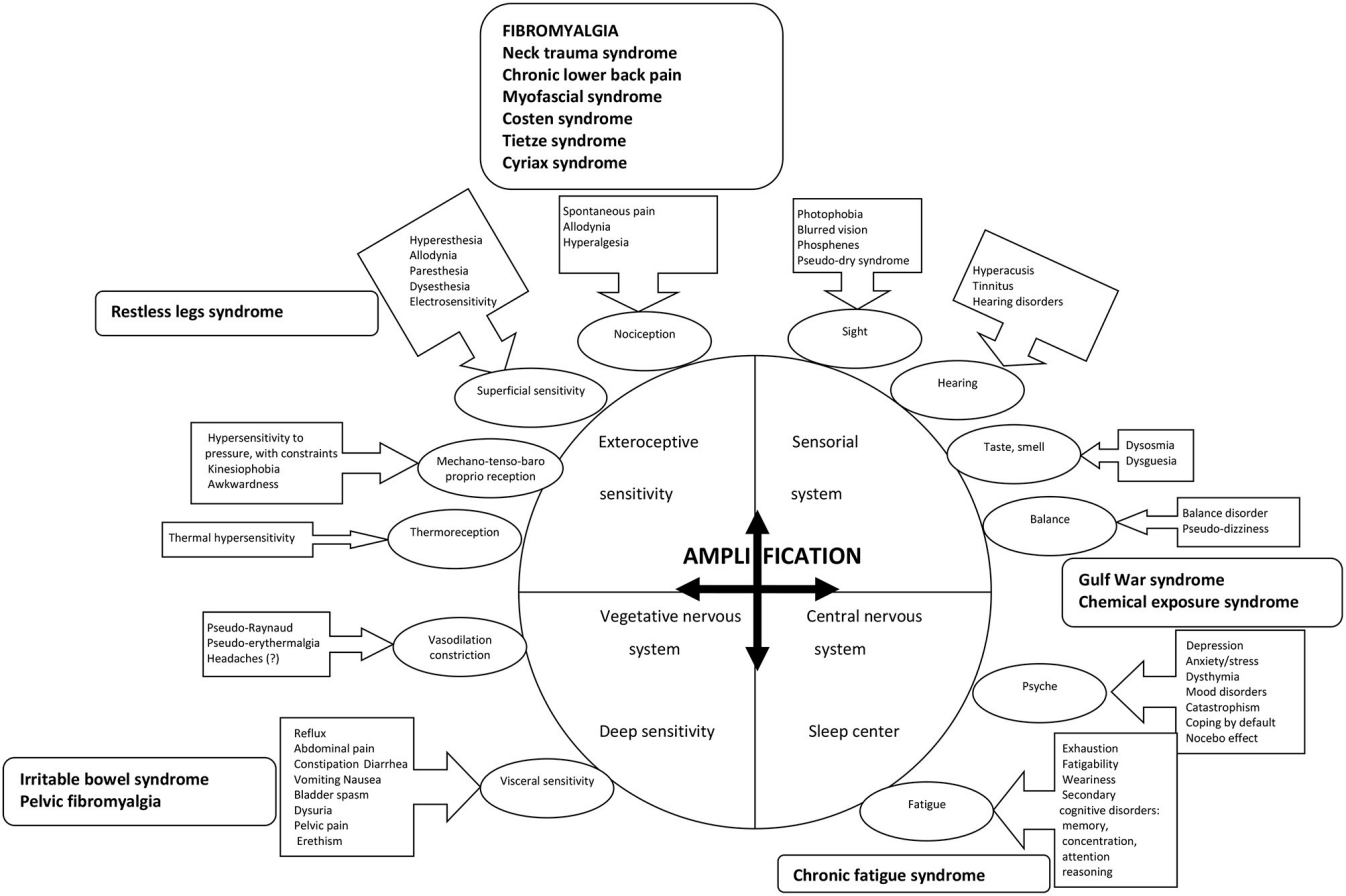
The different paths to the origin of the stimuli responsible for the symptomatology in fibromyalgia are illustrated in the bubbles, with the associated signs in the boxes linked by arrows. The stimuli at the origin of the associated symptoms of fibromyalgia are either of exteroceptive origin, or profound, or related to the neuro-vegetative system, all with medullary relays, or in relation to sensoriality and its bulbar relays, or even central origin with regard to the psyche or fatigue. Each time, there is chronicization and amplification of the signal, which can thus only be done at the central level. The supposed mechanism is either abnormal amplification of the signal itself, or inhibition of the retrocontrol system, or a quantitative decrease in the perception threshold for the signal. A few entities (in bold in the boxes with rounded corners) are the subject of specific names in relation to the more specific predominant symptomatology, but with clear ties with fibromyalgia in the context of the hypersensitization which is common to all these syndromes.

At the basis of this concept of hypersensitization of central origin is the king of fibromyalgia in the 1980s, Yunus (72). Hypersensitization is well-known for pain. As thus are hyperalgesia, allodynia and the famous fibromyalgia pain points from the former criteria. In fact, allodynia and hyperalgesia are generalized. The points that are naturally the most sensitive in anyone are the same in a fibromyalgia patient and are also spontaneously painful. Entheses are structures that are very rich in nociception. But hypersensitization also implies a wide range of other fields, both sensory and psychogenic. Hyperalgesia is thus one criterion that must be taken with a great deal of caution and considered as one of the elements of generalized hypersensitization. Here are types of hypersensitization other than the pain found in fibromyalgia, and which have all been proven (72). Fibromyalgia patients are thus more sensitive to changes in temperature, either hot or cold, and this can be observed when they travel. As has been seen in functional MRI, there is hypersensitization to touch and pressure. Hypersensitization to injections has also been observed when it is necessary to take blood. Patients are also sensitive to ischemia (52). They dislike loud noises and harsh lighting (73). Finally, it has been observed that there is hypersensitization to electricity when an electromyogram is requested. Alongside this sensory hypersensitization, these patients are light sleepers and have side effects to drugs more commonly than other patients treated with the same drugs for other pathologies: at an identical dose, a fibromyalgia patient will have twice as many side effects (74). There is also a high degree of medical failure. It should be noted that all this hypersensitization other than pain can also be found in the sister pathologies to fibromyalgia. Some entities are individualized, with specific denominations, in relation to the more specific predominant symptomatology, as chronic fatigue syndrome, irritable bowel syndrome, neck trauma syndrome, pelvic fibromyalgia, chemical exposure syndrome, gulf war syndrome, myofascial, Costen, Tietze, Cyriax syndromes (Figure 2). Even some presentations of chronic low back pain are sometimes included in this list. All these entities are clear ties with fibromyalgia in a context of the hypersensitization, which is common to all these syndromes. Figure 2 summarizes and collects all the associated symptoms common to the different clinical pictures for hypersensitization. It can involves exteroceptive stimuli or profound sensitivity, or the vegetative nervous system, all with medullary relays. The sensory system will have bulbar relays. The central nervous system itself is involved (fatigue, cognitive, and psychological troubles). The central level is responsible of the chronicization and amplification of all these signals, either by an abnormal amplification of the signal itself, or by inhibition of the retrocontrol system, or a quantitative decrease in the perception threshold for the signal.

## Conclusion

Fibromyalgia is a cognitive disorder of cortical integration of pain. There is amplification of the nociception signal, both at the pain and sensory levels, with a decrease in pain thresholds. There is also persistence of a stimulus, which maintains the condition in a chronicity process. But fibromyalgia must be placed more broadly in the context of chronic hypersensitization syndromes of central origin, a context that is both complex and global, with a very wide range of means of expression.

## Author Contributions

All authors listed have made a substantial, direct and intellectual contribution to the work, and approved it for publication.

## Conflict of Interest

The authors declare that the research was conducted in the absence of any commercial or financial relationships that could be construed as a potential conflict of interest.
